# Small bowel intussusception after Roux-en-Y gastric bypass – A case report

**DOI:** 10.1016/j.ijscr.2025.111193

**Published:** 2025-03-24

**Authors:** Harrison Haeata Keane Gregory, Baillie Ward Churchill Ferris

**Affiliations:** a Department of Surgery, Ipswich Hospital, Chelmsford Avenue, Ipswich, Queensland 4305, Australia; b College of Medicine and Dentistry, James Cook University, 1 James Cook Drive, Douglas, Queensland 4814, Australia

**Keywords:** Case report, Roux-en-Y gastric bypass, Intussusception, Patient management, Surgical intervention

## Abstract

**Introduction and importance:**

Roux-en-Y gastric bypass (RYGB) is a common elective bariatric operation performed to facilitate weight loss in patients with obesity. Although generally associated with a low rate of late complications, intestinal intussusception can very rarely occur in patients who have undergone RYGB, with significant clinical consequences. This case reinforces the importance of prompt recognition and surgical intervention in cases of intussusception following RYGB, explores the proposed pathophysiology of intussusception, and highlights the options for surgical intervention.

**Case presentation:**

We report a case of a 52-year-old female presenting with a small bowel obstruction secondary to intussusception in the context of a RYGB performed 8-years previously. Computed tomography (CT) and diagnostic laparoscopy confirmed intussusception of a long segment of the biliopancreatic limb into the Roux limb through the jejuno-jejunal anastomosis. Unable to be reduced laparoscopically, the anastomosis was resected en bloc and refashioned. The patient recovered well, and reported no features of recurrence or other surgical complication at post-operative review.

**Clinical discussion:**

The aetiology of intussusception following RYGB is unclear, although hypotheses include interruption of duodenal pacemaker cells following transection of the small bowel and consequent development of ectopic jejunal pacemaker cells, increased mobility of the mesentery following extreme weight loss, or the jejunojejunal anastomosis functioning as a transition point. In cases of compromised bowel resection is essential, although the approach in cases of non-compromised bowel is less clear.

**Conclusion:**

The recognition of late RYGB complications is becoming increasingly important in the setting of the increasing prevalence of bariatric surgery. Intussusception is an infrequent but significant complication that requires urgent surgical intervention.

## Highlights

• Small bowel intussusception is an infrequent but clinically significant complication post Roux-en-Y gastric bypass
• Intussusception can occur without a lead point, and in both an anterograde or retrograde fashion
• Hypothesised to occur due to interruption of duodenal pacemaker cells and development of ectopic jejunal pacemaker cells
• Require prompt surgical management, with a role for resection, reduction, plication, or reduction and plication

This work has been reported in line with the SCARE criteria [1].

## 1 Introduction

RYGB was the second-most common bariatric operation in 2023 worldwide, representing 28.5 % of the 449,815 primary bariatric procedures performed [2]. RYGB has a well-established effectiveness in maintaining weight loss and remission from type-2 diabetes mellitis, dyslipidaemia, and hypertension [3]. RYGB involves transection of the stomach from the inferior border of the oblique fat pad to the angle of His, to create a new 20-30 mL gastric pouch. The biliopancreatic (BP) limb, consisting of the remnant stomach in continuity with the duodenum and proximal jejunum, is created by dividing the jejunum 50 to 75 cm from the Ligament of Treitz. The Roux limb is created by measuring 100 to 150 cm from the newly-divided jejunum; the BP limb is anastomosed via a jejunojenunostomy to this point at the distal Roux limb to create a common channel. The proximal end of the Roux limb is then anastomosed via a gastrojejunostomy to the gastric pouch [4]. Intussusception is an exceedingly infrequent cause of small bowel obstruction in patients with RYGB, in which a segment of small bowel invaginates in a retrograde or anterograde nature into the distal or proximal segment causing obstruction. The rate of intussusception in patients with RYGB has been estimated at 0.62 % [5]. Clinical presentation is typically characterised by acute onset generalised abdominal pain and distension, and nausea and vomiting. The mechanism of intussusception, and gold-standard approach to surgical management remains unclear.

## 2 Case background and diagnosis

A 52-year-old Caucasian female presented to the Emergency Department with 2-hour history of acute onset of generalised abdominal pain without a clear precipitant. This was proceeded by profuse nausea, vomiting, and obstipation. The patient had undergone a RYGB 8-years previously with consequent 50 kg of weight loss, and had type-2 diabetes on metformin. On examination, the patient weighed 73 kg, demonstrated normal vital signs, and a generally tender, distended abdomen without evidence of herniation. Bloods were unremarked, apart from a lactate of 4.1. Her pain was poorly controlled with oral oxycodone, sublingual buprenorphine, or intravenous morphine.

**Fig. 1:**
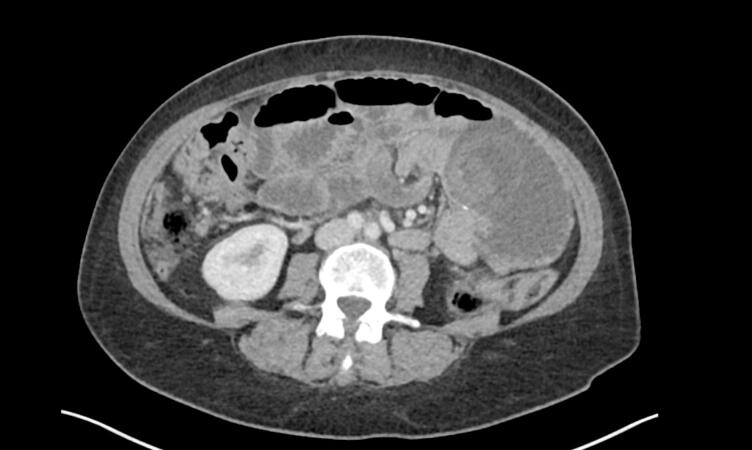
Axial slice of CT abdomen pelvis in the portal venous phase demonstrating high-grade small bowel obstruction with intussusception at the jejunojenunostomy site.

**Fig. 2:**
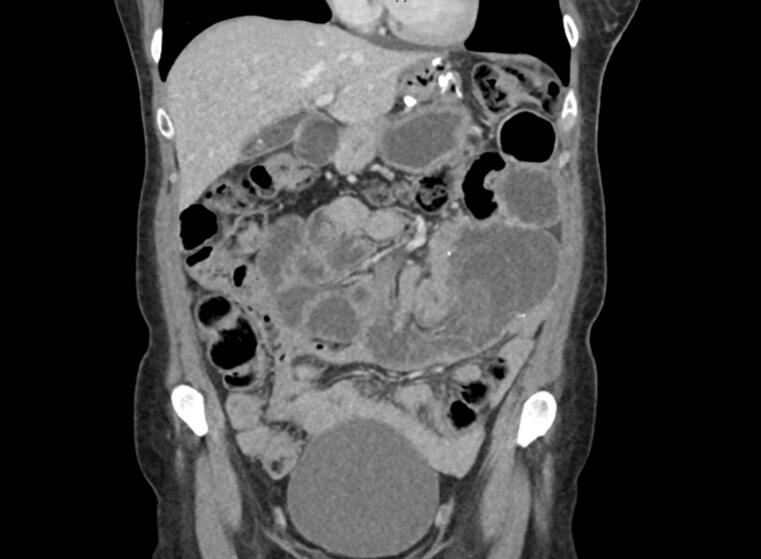
Coronal slice of CT abdomen pelvis in the portal venous phase demonstrating high-grade small bowel obstruction with intussusception at the jejunojenunostomy site in the mid-abdomen.

CT abdomen pelvis with intravenous contrast demonstrated a high-grade small bowel obstruction with a transition point in the mid-abdomen, with appearances suggest of intussusception of the small bowel at the jejunojenuostomy (Fig. 1, Fig. 2). There was also associated swirling of the mesentery of this involved small bowel, and a small volume of abdominal free fluid.

## 3 Treatment

**Fig. 3:**
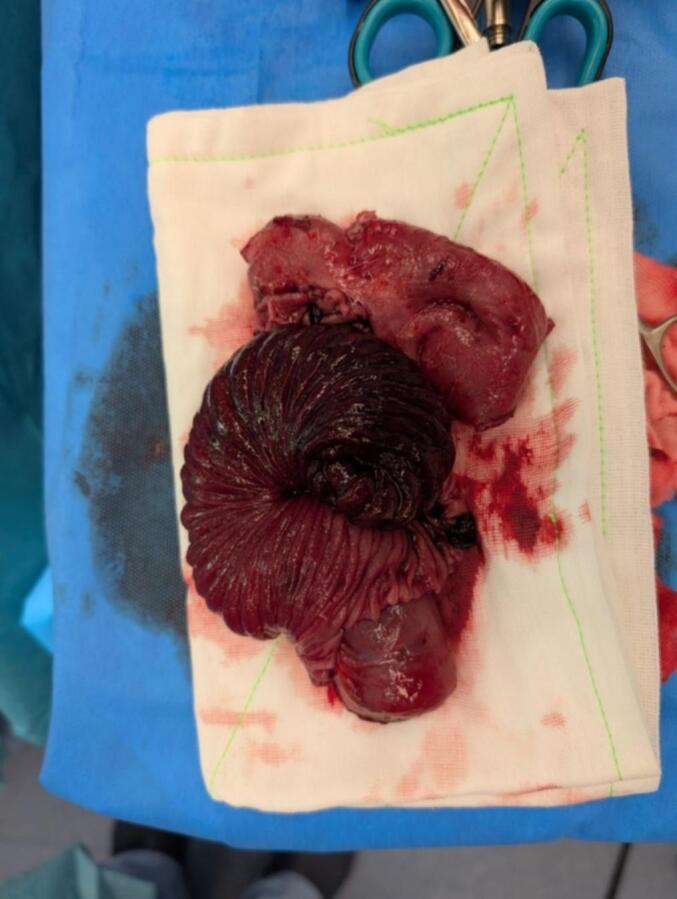
Intraoperative specimen of the resected jejunojejunostomy and involved compromised small bowel.

A nasogastric tube was placed immediately for gastric decompression. The patient proceeded to diagnostic laparoscopy, which revealed a long segment of intussusception of the BP limb into the Roux limb via the jejunojejunostomy causing obstruction (Fig. 3). The involved bowel demonstrated severe venous congestion. This intussusception was unable to be reduced laparoscopically, and the decision was made to convert to open via a mini-midline laparotomy. The jejunojejunostomy and congested bowel was resected en bloc, and the RYGB was reconstructed. No lead point was identified.

## 4 Outcome and follow up

The patient had an uncomplicated post-operative course. Her post-operative abdominal pain was initially controlled with bilateral rectus sheath catheters delivering 0.2 % ropivacaine; she was rapidly de-escalated to sublingual buprenorphine on post-operative day 2. Her nasogastric tube was removed on post-operative day 2, and her bowels opened and diet escalated to regular food on day 3. The patient was discharged on post-operative day 5. 4-week and 6-month follow-up demonstrated no evidence of recurrence of other surgical complication.

## 5 Discussion

Intussusception is an exceedingly infrequent complication of RYGB. Commonly, intussusception is a pathology seen in children aged 3-months to 3-years. In this age group, 90 % of cases are idiopathic and 5 % are secondary to a pathological lead point such as intestinal polyps, lymphoid hyperplasia, lymphoma or Meckel's diverticulum [5]. Intussusception is less common an entity in adults, and is much more commonly associated with a pathological lead point in this population (∼90 %) [6]. Contrarily, lead points are infrequent in RYGB patients with intussusception [5,6]. As opposed to patients without RYGB, where intussusception more commonly occurs in an anterograde fashion, patients with RYGB demonstrate retrograde intussusception more often [5]. The exact mechanism of intussusception in RYGB is unclear. Studies suggest that during formation of the Roux limb, the transection of the jejunum separates the distal small bowel from the duodenal pacemaker cells which initiate coordinated intestinal mobility [[7], [8], [9]]. Consequently, ectopic pacemaker cells develop in the jejunum of the Roux limb, resulting in discontiguous peristalsis. As a result, anterograde peristaltic waves from the duodenal pacemaker cells may meet retrograde peristaltic waves from the ectopic jejunal pacemaker cells, resulting in intussusception [[7], [8], [9]]. Other hypotheses include the increased mobility of the mesentery surrounding the jejunojejunal anastomosis with extreme weight loss; and the jejunojejunal staple-line anastomosis acting as a lead point. A case series including 665 patients by Orthopoulos et al. demonstrated that all patients who developed intussusception had a jejunojejunostomy length greater that 60 mm [10].

Given the compromised nature of the intussuscepted bowel in this case, our operative approach involving resection was congruent with suggested management in the literature. In a meta-analysis of intussusception following RYGB by Oor et al., all 107 patients with identified intussusception proceeded to surgical exploration, and a majority (78 %) started with a laparoscopic approach [4]. In this meta-analysis, four surgical approaches were identified – reduction, plication, reduction and plication, and resection. In all studies, patients with compromised bowel proceeded to resection. In cases where bowel is not compromised, an avoidance of resection predominates. Interestingly, rates of recurrence of intussusception ranged from 3.6 % to 26.5 % in Oor's meta-analysis, with the highest rate of recurrence associated with patients who underwent reduction alone [4]. As such, the risk of recurrence must be balanced with the consequences of resection on small bowel function and surgical risk associated with refashioning of the RYGB anastomoses.

## 6 Limitations

This case study reports on the approach and outcomes in a single patient presenting with intussusception following RYGB. A larger population of patients presenting with intussusception would be beneficial to compare patient outcomes and varying surgical approach.

## 7 Conclusion

RYGB was the second-most common bariatric operation in 2023 worldwide, representing 28.5 % of the 449,815 primary bariatric procedures performed. As an increasing number of patients undergo RYGB, an awareness of infrequent but potential complications is essential. Intussusception is an example of these infrequent complications, and without prompt surgical intervention can result in serious harm or death. Intraoperatively resection of compromised bowel is required, although consideration of resection versus reduction, or reduction and plication in cases without bowel compromise is reasonable if the risk of recurrent intussusception is acknowledged.

## Author contribution

Harrison Gregory – case analysis, writing, perioperative care

Baillie Ferris – supervision, perioperative care

## Consent

Written informed consent was obtained from the patient for publication of this case report and accompanying images. A copy of the written consent is available for review by the Editor-in-Chief of this journal on request.

## Ethical approval

As per the West Moreton Hospital and Health Service which oversees Ipswich Hospital, a case report is exempt from ethical approval

## Guarantor

Harrison Gregory.

## Research registration number

Research is not ‘first in man’ study – therefore no registration required.

## Funding

N/A.

## Conflict of interest statement

N/A.
